# A Literal Investigation Into the Existence of Leiomyomatous Erythrocytosis and Secretion of Erythropoietin-Like Substances: A Systematic Review

**DOI:** 10.7759/cureus.111656

**Published:** 2026-06-28

**Authors:** Adeola O Adegunle, Itunujesu J Olonade, Bryan Felix, Suma Kaza

**Affiliations:** 1 Obstetrics and Gynecology, Avalon University School of Medicine, Willemstad, CUW

**Keywords:** angiogenesis, erythrocytosis, erythropoietin, leiomyoma, secondary polycythemia

## Abstract

Uterine leiomyomas, or fibroids, are a very frequent benign tumor in women of reproductive age. Erythrocytosis is an increase in the number of red blood cells, leading to polycythemia. Erythrocytosis as a paraneoplastic condition associated with leiomyomas is an area not well studied. This study aimed to investigate the production of erythropoietin-like substances in uterine leiomyomas and their association with secondary erythrocytosis, as well as to identify additional symptoms and complications beyond those traditionally associated with leiomyomas.

A literature search was conducted using PubMed and EBSCO for articles published between 2009 and 2023. Eligible studies included case reports and clinical studies involving patients aged ≥25 years diagnosed with uterine leiomyoma complicated by polycythemia. Hematologic criteria for polycythemia and pathological evidence of erythropoietin or erythroid activity within the tumor were analyzed. Study selection and eligibility assessment were performed independently by two reviewers.

Thirteen studies met the inclusion criteria, comprising 1 clinical study, 1 case series, and 11 case reports. Among the case reports, 11 described single cases, while one included 3 cases, and the clinical study involved 114 participants. All included cases demonstrated polycythemia in association with uterine leiomyoma, with a positive correlation observed between tumor size and erythrocytosis. Hematologic findings consistently showed elevated hemoglobin, hematocrit, erythropoietin levels, and increased red blood cell counts. Histopathological analyses, based on specimens obtained via tumor resection, hysterectomy, or salpingectomy, supported the presence of erythropoietin production within the leiomyomas. These findings are consistent with myomatous erythrocytosis syndrome (MES), a rare but well-documented condition.

MES is not a common paraneoplastic syndrome of leiomyoma, but it is still an occurrence that has to be taken note of to avoid unnecessary testing. Future research should prioritize prospective studies systematically screening women with leiomyomata for erythrocytosis and the time of diagnosis; matched case-control studies comparing EPO expression and hematologic profiles between women with MES and those with leiomyoma alone; molecular studies characterizing the mechanisms of EPO-independent erythrocytosis in leiomyoma; and the development of clinical guidelines for the evaluation and management of MES-affected women.

## Introduction and background

Introduction

Uterine leiomyomas, or fibroids, are very frequent benign tumors in women of reproductive age. Despite their benign nature, they can have a substantial impact on the daily physical and mental well-being of women with this condition [1]. The predicted incidence of fibroids in women by the age of 50 was 70% for Caucasian women and more than 80% for Black women [2]. Though the exact pathophysiology of fibroids is still poorly understood, they are said to be formed by uterine smooth muscle cells, and their proliferation is mostly determined by circulating estrogen levels. Fibroids can appear as an asymptomatic incidental observation on imaging or be symptomatic [2]. Typically, they are identified through physical examination and ultrasound imaging, which has a high sensitivity for this condition. Fibroids continue to be the major reason for hysterectomy, as leiomyomata account for 39% of all hysterectomies performed annually [3]. Some of the most common symptoms are irregular uterine hemorrhage, pelvic pain, disruption of adjacent pelvic tissues (bowel and bladder), back pain, and erythrocytosis.

Erythrocytosis is characterized by an increase in the number of red blood cells in the body, usually a common incidental abnormality found during laboratory testing that demonstrates sustained elevations in hematocrit levels of >52% in males and >48% in women [4]. In many cases, erythrocytosis is the result of an underlying cardiovascular condition, an androgen-induced medication reaction, or smoking, rather than a primary bone marrow disorder like polycythemia vera [4]. Erythrocytosis can be divided into primary and secondary erythrocytosis. Primary erythrocytosis is caused by polycythemia vera, a myeloproliferative neoplasm in which aberrant bone marrow cells create an excess of red blood cells, as well as white blood cells and platelets; rarely, only red blood cell synthesis is increased without the increase of other hematologic lines [5]. Secondary erythrocytosis, on the other hand, typically occurs as a result of elevated erythropoietin levels, a hormone produced by the kidneys that stimulates bone marrow to create red blood cells. Some of these conditions include anemia and malignancies [5].

Many evaluations have been conducted on the various forms of leiomyomas and how they affect symptom presentation and illness prognosis [6], but an area less assessed is erythrocytic paraneoplastic disease, a less common but possible complication of leiomyoma. It is thought that leiomyomas create erythropoietin-like elements, which cause polycythemia in women with leiomyomas. Multiple cases where the resolution of polycythemia symptoms following hysterectomy in women with fibroids have been reported [7]. Seeing as this complication is less studied but still presents and could affect the course of assessment and management of leiomyomas, we thought to take a look at available data to review possible manifestations of this phenomenon, as well as possible underlying factors contributing to the development of erythrocytosis by looking at different hematologic parameters and available imaging or other diagnostic tools employed.

Objectives

The objectives of this study were to review possible sources of erythrocytosis and the parameters to look out for to better assess and manage leiomyoma uteri, and to establish new symptoms and complications that can be seen in leiomyoma aside from pre-existing complications.

## Review

Methods

Search Strategy

A comprehensive literature search was conducted on 9 June 2024 using the PubMed and EBSCO** **databases to identify studies investigating the association between uterine leiomyomas, erythropoietin, and myomatous erythrocytosis syndrome (MES). 

The search strategy combined vocabulary terms (Medical Subject Headings [MeSH] in PubMed and CINAHL/EBSCO subject headings) with free-text keywords to maximize the retrieval of relevant publications. The search incorporated terms related to four key concepts: uterine leiomyomas (uterine fibroids); erythropoietin (EPO); polycythemia/erythrocytosis, including myomatous erythrocytosis syndrome (MES); and women or female subjects. Boolean operators (AND and OR) were used to combine synonyms within each concept and to integrate the different concepts into a comprehensive search strategy. 

The PubMed search included the terms "Leiomyoma," "Leiomyomata," "uterine fibroid," "uterine myoma," "Erythropoietin," "EPO," "Polycythemia," "erythrocytosis," and "Myomatous Erythrocytosis Syndrome," together with the MeSH terms "Leiomyoma," "Erythropoietin," "Polycythemia," and "Women/Female." The EBSCO search employed the corresponding subject headings and equivalent free-text terms. Both database searches were restricted to English-language articles published between 1 January 2009 and 31 December 2023. The search yielded 27 records from PubMed and 13 records from EBSCO, resulting in 40 records before removal for eligibility and other reasons. The complete electronic search strategies for both databases are provided below.

Study Selection and Eligibility Criteria (Inclusion and Exclusion)

Full-text articles were obtained for the citations that met the abstract and title screening criteria based on our PICO (Population, Intervention, Comparison, Outcome) concepts. The study was a systematic review of case reports and clinical studies of myomatous polycythemia as a result of erythropoietin-like products. The research population focused on women of reproductive age, that is, women in the age range of 25-60. For articles to be included, the full-text screening had to mention the following: i) Women with leiomyoma, ii) Women age >25 years, iii) Erythropoietin-like product, iv) Erythrocytosis or polycythemia.

Articles were excluded based on the following criteria: i) population other than women with leiomyoma; ii) participant age <25 years; iii) Outcomes did not include erythrocytosis or polycythemia; iv) Pre-existing conditions of polycythemia.

Measurement and Observation: Outcome, Exposures, Covariates

After screening articles by title and abstract identified by the search strategy, we extracted 40 potentially relevant articles. Of the 40 articles, we excluded 27 articles if they did not meet our inclusion criteria, did not include erythrocytic outcomes, were a protocol study for a clinical study, specified polycythemia for pre-leiomyoma and not during the presence of the tumor, or did not exclude previous hematologic diagnoses that could potentially be a confounding factor. 

Data Extraction

We prepared a data extraction form and tested it using six articles. In the data extraction form, full-text articles meeting the inclusion and exclusion criteria were analyzed for descriptive data, such as study design, location, and population characteristics, and clinical outcomes such as hematologic parameters, histopathology, and intervention efficacy. We proceeded to extract descriptive data (ethical approval, study period, control data, confounding factors, limitations, percentage lost to follow-up, and methodological limitations) and participant data (age, race, BMI, weeks of presence of tumor, diagnostic criteria, and baseline measures); interventions (type, frequency, duration, dose, route of administration, and duration of follow-up); measures of frequency (number of participants, incidence, and prevalence); and outcomes (erythrocyte count, hemoglobin levels, polycythemic manifestations, change in blood viscosity, and other hematologic manifestations). Disagreements were resolved by consensus, and the relevant data were subsequently extracted.

Data Analysis

Given the heterogeneity of the included studies, comprising 1 clinical study and 12 case reports with variable sample sizes, geographic settings, and outcome reporting, a formal meta-analysis was not performed; instead, we performed a descriptive synthesis of the available data. For hematologic outcomes reported across multiple studies (hemoglobin, hematocrit, erythropoietin, and red blood cell count), we calculated arithmetic means and ranges from individually reported values. These figures are presented for descriptive purposes only and should not be interpreted as pooled estimates. Due to the predominance of case reports in the included literature, incidence and prevalence data could not be reliably estimated; figures reported by original authors are described qualitatively where available.

Quality and Risk-of-Bias Assessment

The methodology quality of included case reports was assessed independently by two reviewers (AA, IO) using the Joanna Briggs Institute (JBI) Critical Appraisal Checklist for case reports, an eight-item validated tool that evaluates: (1) patient demographics, (2) patient history, (3) clinical condition at presentation, (4) diagnostic testing, (5) interventions, (6) post-intervention condition, (7) adverse events, and (8) takeaway lessons. Items were scored as 'Yes' (met), ‘No’ (not met), or ‘Unclear’ (insufficient information), and a total score out of 8 was derived for each case report. The single clinical study was appraised using the National Heart, Lung, and Blood Institute (NHLBI) Quality Assessment Tool for Observational Cohort and Cross-Sectional Studies, which examines items including selection bias, measurement or exposure and outcome, blinding, and follow-up completeness. Disagreements between reviewers were resolved by discussion and consensus. Overall study quality was rated as high (≥7/8 JBI met), moderate (5-6/8), or low (≤4/8). These ratings were considered when interpreting the strength of the overall evidence base but were not used as grounds for exclusion, given the scarcity of primary literature on this topic. Publication bias was assessed informally; given that the majority of included studies are case reports, a formal funnel plot analysis was not appropriate.

Results

Description of Studies Included

**Figure 1:**
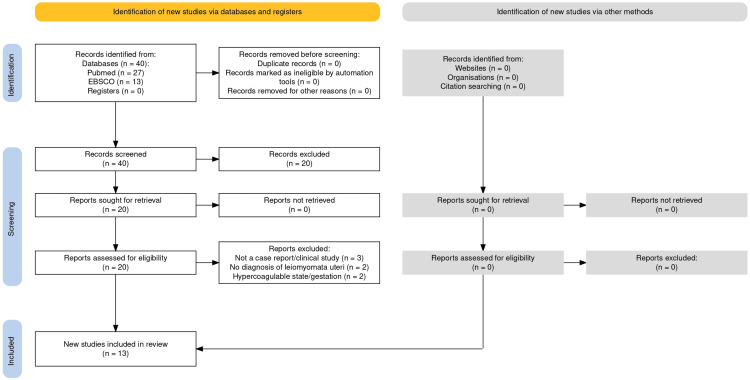
Breakdown of included and excluded studies made using PRISMA 2020 guidelines Source: [8]

Forty articles were identified during the literature search. Twenty were excluded based on the title and abstract, leaving 20 to be included as potentially relevant articles. Three articles were excluded further during data extraction for not being a case report or clinical study, two for not including a diagnosis of Leiomyomata uteri, and another two due to the presence of other conditions leading to a hypercoagulable state, such as gestation. The final analysis included 13 articles, as described in Figure 1.

Study Characteristics

Of the 13 studies included, 1 was a clinical study [7], and the 12 others were case reports. Eleven of the case reports had one case each, while one looked at three cases [9]. The only clinical study had 114 participants. Four studies were carried out in Japan [7,9-12], three in the USA [13-16]; three were set in Europe (Belgium, the Netherlands, and the UK) [15,17,18], and three in Asia (the Philippines, Thailand, and China) [9,19,20]. All the studies included women diagnosed with Leiomyomata uteri (Table 1). No study included women who were less than 25 years of age or had no diagnosis of leiomyoma. All studies were carried out after 2009, except for one carried out in 2008.

**Table 1:** Characteristics of studies included in this review of case reports and clinical study data extraction Epo: erythropoietin

Study	Study design & location	Participant	Sample characteristics	Interventions	Outcomes measured
Guzman, 2019 [9]	Case report, Philippines	3	All caucasian, cases 1-46, 2-45, and 3-27	Surgical resection	Hb, Hematocrit, Epo
Couillandre, 2022 [17]	Case report, Belgium	1	34-year-old caucasian woman	Surgical resection of tumor	Hb, Hematocrit, Epo
Thiangphak, 2023 [19]	Case report, Thailand	1	56-year-old asian woman	Surgical resection	Hb, Hematocrit, Red cell count
Asano, 2015 [7]	Clinical Study, Japan	114	Asian women with age ranging from 25 to 71	Hysterectomies and surgical resection	Hb, Hematocrit, Epo
Ono, 2014 [10]	Case report, Japan	1	55-year-old Asian woman	hysterectomy	Hb, Hematocrit, Red cell count
Vlasveld, 2008 [18]	Case report, Netherlands	1	45-year-old Caucasian woman	Hysterectomy	Hb, Hematocrit, Red cell count
Suresh, 2019 [14]	Case report, USA	1	49-year-old Caucasian woman	Surgical resection	Hb, Hematocrit, Epo, Red cell count
Shu, 2021 [20]	Case report, China	1	47-year-old Asian woman	Hysterectomy and bilateral salpingectomy	Hb, Hematocrit
Unosawa, 2009 [11]	Case report, Japan	1	53-year-old Asian woman	Hysterectomy and bilateral salpingectomy	Hb, Hematocrit, Epo, Red cell count
Narita, 2015 [12]	Case report, Japan	1	39-year-old Asian woman	total hysterectomy	Hb, Hematocrit, Epo, Red cell count
Pavadala, 2010 [15]	Case report, UK	1	51-year-old Caucasian woman	Hysterectomy and bilateral salpingo-oophrectomy	Hb, Hematocrit, Epo, Red cell count
Valente, 2024 [13]	Case report, USA	1	44-year-old Caucasian woman	Hysterectomy and bilateral salpingectomy	Hb, Hematocrit, Epo, Red cell count
Gordon, 2019 [16]	Case report, USA	1	68-year-old African American woman	Uterine artery embolization	Hb, Hematocrit, Epo

Hematologic Parameters of Studies

All 13 of the cases included measurements of hemoglobin and hematocrit levels. Nine cases included measurement of erythropoietin levels [7,9,11-17], and eight measured erythrocyte count [10-15]. Only five of the case reports were symptomatic [11-13,15,16]; the remaining eight were asymptomatic and did not have any physical manifestations of polycythemia. None of the cases had the presence of other hematologic conditions, with platelet and white blood cell levels being normal.

Case Outcomes

The study outcomes included hemoglobin levels, erythropoietin levels, hematocrit value, and red blood cell count, as shown in Table 2. All the cases had elevated hemoglobin levels ranging from above 17g/dl [11] to below 25 g/dl [15], with a mean of 19 g/dl. Hematocrit levels across the cases ranged from 52.5% [12], the lowest value, to 66%, the highest [15], with a mean of 61.7%. Eight cases measured erythropoietin levels. Going by the normal range of erythropoietin being 2.6 to 18.5 mU/mL, 7 out of 8 had elevated erythropoietin levels, ranging from 19.3 mU/mL [12] to 49 mU/mL [15] and a mean of 38.1 mU/mL. The remaining one case report had a normal erythropoietin level of 5.7 mU/mL [17]. The clinical study with 15 participants had a mean erythropoietin level of 32.5 mU/mL [7]. Seven of the cases measured red blood cell count with values ranging from 5.3 m cells/ul [11] to 7.9 m cells/ul [15] and a mean of 6.37m cells/ul.

**Table 2:** Pooled data of hematologic outcomes in the included studies Epo: erythropoietin

Study	Outcomes	Polycythemic manifestations
Guzman, 2019 [9]	Hb- mean of 19.1 g/dl; Hematocrit- mean of 57.7%; Epo- mean of 24.3 mU/ml	None
Couillandre, 2022 [17]	Hb- 19.9 g/dl; Hematocrit- 60.5%; Epo- 5.7 mU/ml	None
Thiangphak, 2023 [19]	Hb-18.1 g/dl; Hematocrit- 56.1%; Red cell count- 6.52 m cells/ul	None
Asano, 2015 [7]	Hb- mean of 19.4 g/dl; Hematocrit- mean of 60.2%; Epo- mean of 32.6 mU/ml	None
Ono, 2014 [10]	Hb- 19.9 g/dl; Hematocrit- 59.1%; Red cell count- 6.65 m cells/ul	None
Vlasveld, 2008 [18]	Hb- 17.2 g/dl; Hematocrit- 53%; Red cell count- 5.69 m cells/ul	None
Suresh, 2019 [14]	Hb- 17.6 g/dl; Hematocrit- 54.3%; Epo- 24.6 mU/ml; Red cell count- 5.75 m cells/ul	None
Shu, 2021 [20]	Hb- 19.7 g/dl; Hematocrit- 58.2%	None
Unosawa, 2009 [11]	Hb- 18.1 g/dl; Hematocrit- 53.5%; Epo- 29.3 mU/ml; Red cell count- 5.3 m cells/ul	Thromboembolism leading to pulmonary embolism, dyspnea
Narita, 2015 [12]	Hb- 18 g/dl; Hematocrit- 52.7%; Epo- 19.3 mU/ml; Red cell count- 5.86 m cells/ul	General fatigue, dyspnea, and headache
Pavadala, 2010 [15]	Hb- 23 g/dl; Hematocrit- 66%; Epo- 49 mU/ml; Red cell count- 7.9 m cells/ul	Discolored finger, tiredness, lack of concentration
Valente, 2024 [13]	Hb- 19.4g/dl; Hematocrit- 59.3%; Epo- 22.2 mU/ml; Red cell count- 6.8m cells/ul	Headache, fatigue, dyspnea due to PE, Venous sinus thrombosis.
Gordon, 2019 [16]	Hb- mean of 19.8g/dl; Hematocrit- mean of 62.4%; Epo- mean of 31.1 mU/ml	Intermittent dizziness, right arm numbness

Only five out of the cases reported physical manifestations of polycythemia; three had symptoms such as headache and fatigue [12,13,15], one experienced dizziness [16], and three had thromboembolic events, such as venous sinus thrombosis and pulmonary embolism, leading to dyspnea and chest pain [11,13]. Other symptoms included lack of concentration, discolored fingers, and arm numbness, which could be attributed to hyperviscosity or thromboembolism [15,16].

For the interventions carried out, seven of the cases opted for hysterectomies [7,10,11,15,18,20]. Of the seven, two were hysterectomies plus bilateral salpingectomy [11,20], while the other two were hysterectomies plus bilateral salpingo-oophorectomy [13,15]. Four cases were treated with surgical resection of the tumors, and for one case, uterine artery embolization was carried out [16]. In the clinical study, interventions included hysterectomies and surgical resection [7]. All the cases showed rapid resolution of polycythemia after interventions were carried out.

Of the 13 studies included in this review, the majority were case reports (n=11), with one case series [9] and one clinical study [17], reflecting the rarity of the condition and the early stage of the evidence base. Quality appraisal using JBI/NHLBI criteria yielded six studies rated as high quality and seven as moderate quality, with no studies rated as low [21,22]. Erythropoietin was measured in 12 of the 13 studies, with Thiangphak 2023 failing to report this parameter. Confounder management was the most variable domain: six studies fully addressed confounders, four partially addressed them, and three did not report this clearly, as seen in Table 3. Histopathology was performed in 10 of the 13 studies, with three being the exception [13,12,16]. Overall, the evidence base demonstrates reasonable methodological quality given the inherent limitations of case-level study designs, though incomplete confounder control and the absence of higher-level study designs remain important limitations.

**Table 3:** Quality and risk assessment of the pooled data using the JBI and NHLBI checklist Epo: erythropoietin; JBI: Joanna Briggs Institute [21]; NHLBI: National Heart, Lung, and Blood Institute [22]

Study	Design	JBI / NHLBI Score	Quality Rating	Epo measured?	Confounders	Histopathology performed
Guzman, 2019 [9]	Case Series	7/10	Moderate	Yes	Partial	Yes
Couillandre, 2022 [17]	Case Report	8/8	High	Yes	Yes	Yes
Thiangphak, 2023 [19]	Case Report	6/8	Moderate	No	Partial	Yes
Asano, 2015 [7]	Clinical Study	10/14	Moderate	Yes	Yes	Yes
Ono, 2014 [10]	Case Report	7/8	High	Yes	Yes	Yes
Vlasveld, 2008 [18]	Case Report	7/8	High	Yes	Yes	Yes
Suresh, 2019 [14]	Case Report	8/8	High	Yes	Yes	Yes
Shu, 2021 [20]	Case Report	6/8	Moderate	Yes	Partial	Yes
Unosawa, 2009 [11]	Case Report	6/8	Moderate	Yes	Partial	Yes
Narita, 2015 [12]	Case Report	5/8	Moderate	Yes	Yes	No
Pavadala, 2010 [15]	Case Report	7/8	High	Yes	Yes	Yes
Valente, 2024 [13]	Case Report	7/8	High	Yes	Yes	No
Gordon, 2019 [16]	Case Report	6/8	Moderate	Yes	Yes	No

Discussion

Principal Finding

Cases demonstrating polycythemia outcomes for women diagnosed with leiomyoma or uterine fibroids were identified. Elevated hemoglobin levels, increased hematocrit levels, elevated erythropoietin levels, a high red cell count, and polycythemia-like physical manifestations were among the hematologic outcomes of interest. After reviewing the data of the 12 case reports and clinical studies that satisfied our inclusion criteria, we determined that women with leiomyomas have an elevated risk of polycythemia due to myomatous erythrocytosis syndrome. In our review, we discovered that removing the benign tumors resulted in the resolution of existing polycythemia. Hematologic readings returned to normal quickly, with the majority of individuals reporting a decline in hemoglobin levels as low as 12.6 at 24 hours after surgery [14].

Relationship Between Erythrocytosis and Leiomyoma

Many of the cases also examined the connection between leiomyoma and erythrocytosis. A few cases used staining techniques that showed the expression of erythropoietin-producing cells (Epo-R) in the myoma [18]. Despite the fact that the precise relationship is still unknown, they all suggest that the polycythemia observed can be attributed to the production of erythropoietin by the cells of the myoma [7]. Some patients reported increased erythropoietin production in the uterine artery, which resulted in enhanced angiogenesis in the myoma. The increased angiogenesis is assumed to be the reason why myomatous erythrocytosis syndrome cases were found in women with very large and highly vascularized tumors [15]. This association is also shown in the report by Narita 2015, where intervention with uterine artery embolization resulted in a decrease in both erythrocytosis and tumor size [16].

Clinical Significance and Diagnostic Implications

The rarity of MES creates a significant risk of diagnostic delay. As illustrated by one included case, a patient experienced polycythemia for over six years before the association with her underlying leiomyoma was recognized. In clinical practice, women presenting with unexplained absolute erythrocytosis, particularly in the absence of cardiopulmonary disease, myeloproliferative neoplasm, or smoking history, should be assessed for the presence of pelvic tumors, including uterine leiomyomata, as part of the secondary erythrocytosis workup. Awareness of MES is especially important given the potentially serious thromboembolic sequelae observed in three of the included cases, including venous sinus thrombosis and pulmonary embolism. The uniformly rapid resolution of polycythemia following surgical or interventional management of the fibroid further supports a causal relationship between the two conditions and has direct implications for treatment decision-making.

Limitations

This review has several limitations that must be acknowledged. First, the review is dominated by case reports, 12 out of 13 included studies, representing the lowest level of individual study evidence, and making it susceptible to reporting bias. It is likely that cases of MES in which polycythemia was not detected or documented were not published, inflating the apparent association. Second, there was substantial variation in the measurement and reporting of hematologic outcomes across studies, with five studies not reporting serum erythropoietin [10,16,18-20] and five not reporting red cell count [7,9,16,17,20], limiting comparability. Another limitation was the lack of diversity in the geographic and demographic distribution of the cases, with Asian and Caucasian women being the predominant races in the studies reviewed, which may tend to limit generalizability. Last, this review did not identify any studies that directly compared women with MES to those with leiomyoma in the absence of erythrocytosis, making it impossible to characterize risk factors for developing MES among women with fibroids.

The review had its strengths, with quite a number of studies measuring all parameters of polycythemia as well as performing staining techniques to fully understand the pathophysiology of this phenomenon [11-15].

## Conclusions

Myomatous erythrocytosis (MES) is not a common paraneoplastic syndrome of leiomyoma, but it is still an occurrence that has to be taken note of to avoid unnecessary testing. Future research should prioritize prospective studies systematically screening women with leiomyomata for erythrocytosis and the time of diagnosis; matched case-control studies comparing erythropoietin expression and hematologic profiles between women with MES and those with leiomyoma alone; molecular studies characterizing the mechanisms of erythropoietin-independent erythrocytosis in leiomyoma; and the development of clinical guidelines for the evaluation and management of MES, including the role of uterine artery embolization as a fertility-preserving alternative to hysterectomy in affected women.

## References

[1] Barjon K, Mikhail LN (2025). Uterine leiomyomata. StatPearls [Internet].

[2] Baird DD, Dunson DB, Hill MC, Cousins D, Schectman JM (2003). High cumulative incidence of uterine leiomyoma in black and white women: ultrasound evidence. Am J Obstet Gynecol.

[3] De La Cruz MS, Buchanan EM (2017). Uterine fibroids: diagnosis and treatment. Am Fam Physician.

[4] Hodges VM, Rainey S, Lappin TR, Maxwell AP (2007). Pathophysiology of anemia and erythrocytosis. Crit Rev Oncol Hematol.

[5] Liesveld JL (2025). Secondary erythrocytosis. Merck Manual Consumer Version.

[6] Okolo S (2008). Incidence, aetiology and epidemiology of uterine fibroids. Best Pract Res Clin Obstet Gynaecol.

[7] Asano R, Asai-Sato M, Miyagi Y (2015). Aberrant expression of erythropoietin in uterine leiomyoma: implications in tumor growth. Am J Obstet Gynecol.

[8] Haddaway NR, Page MJ, Pritchard CC, McGuinness LA (2022). PRISMA2020: an R package and Shiny app for producing PRISMA 2020-compliant flow diagrams, with interactivity for optimised digital transparency and open synthesis. Campbell Syst Rev.

[9] de Guzman GS, Manalo EM (2019). Myomatous erythrocytosis syndrome: a case series. Case Rep Womens Health.

[10] Ono Y, Hidaka T, Fukuta K, Kouchi K, Yasoshima K, Takagawa K, Arai T (2014). A case of myomatous erythrocytosis syndrome associated with a large uterine leiomyoma. Case Rep Obstet Gynecol.

[11] Unosawa S, Hata M, Sezai A, Niino T, Yoshitake I, Minami K (2009). Pulmonary embolism with myomatous erythrocytosis syndrome and extreme obesity. Thorac Cardiovasc Surg.

[12] Narita F, Ohara N, Fukunaga K (2003). Myomatous erythrocytosis syndrome. J Obstet Gynaecol.

[13] Valente E, Zueger M, Donato D (2024). A rare case of venous sinus thrombosis and pulmonary embolisms secondary to myomatous erythrocytosis syndrome. AJOG Glob Rep.

[14] Suresh P, Rizk S (2020). Myomatous erythrocytosis syndrome: case report and review of the literature. Cureus.

[15] Padavala J, Abdelmagied A, Emery S (2010). Rapidly developing myomatous erythrocytosis syndrome: a case report. BMJ Case Rep.

[16] Gordon B, Fischbeck T, Salamo R, Schroff S (2019). Treating myomatous erythrocytosis syndrome with uterine artery embolization. Obstet Gynecol.

[17] Couillandre P, Lewalle P, Benghiat SF, Salaroli A (2023). Surprising erythrocytosis resolution after myomectomy: myomatous erythrocytosis syndrome. Eur J Case Rep Intern Med.

[18] Vlasveld LT, de Wit CWM, Verweij RA, Castel A, Jansen PM, Peters AAW (2008). Myomatous erythrocytosis syndrome: further proof for the pathogenic role of erythropoietin. Neth J Med.

[19] Thiangphak E, Jiamset I, Matemanosak P, Rattanaburi A (2023). Secondary erythrocytosis associated with uterine myoma is rare but should be of concern. Case Rep Obstet Gynecol.

[20] Shu XY, Chen N, Chen BY, Yang HX, Bi H (2022). Myomatous erythrocytosis syndrome: a case report. World J Clin Cases.

[21] Aromataris E, Fernandez R, Godfrey C, Holly C, Kahlil H, Tungpunkom P (2015). Summarizing systematic reviews: methodological development, conduct and reporting of an umbrella review approach. Int J Evid Based Healthc.

[22] (2026). National Heart, Lung, and Blood Institute. Quality assessment of systematic reviews and meta-analyses. https://www.nhlbi.nih.gov/health-topics/study-quality-assessment-tools.

